# An integrated database of *Eucalyptus* spp. genome project

**DOI:** 10.1186/1753-6561-5-S7-P170

**Published:** 2011-09-13

**Authors:** Leandro Costa Nascimento, Jorge Lepikson Neto, Marcela Mendes Salaza, Eduardo Leal Oliveira Camargo, Wesley Leoricy Marques, Danieli Cristina Gonçalves, Ramon Oliveira Vidal, Gonçalo Amarante Guimarães Pereira, Marcelo Falsarella Carazzolle

**Affiliations:** 1 Laboratório de Genômica e Expressão - Instituto de Biologia - Universidade Estadual de Campinas – UNICAMP, Brazil; 2 Laboratório de Genômica e Expressão - Instituto de Biologia - Universidade Estadual de Campinas - UNICAMP/LNBio - Laboratório Nacional de Biociências – ABTLuS, Brazil; 3 Laboratório de Genômica e Expressão - Instituto de Biologia - Universidade Estadual de Campinas - UNICAMP/Centro Nacional de Processamento de Alto Desempenho em São Paulo, Universidade Estadual de Campinas-UNICAMP, Brazil

## Background

The species of the genus *Eucalyptus* are the most planted for the fiber crop in the world. They are mainly utilized for timber, pulp and paper production. Brazil, helped by the favorable weather conditions, appears as a big producer and exporter of eucalyptus derivates. In 2002, the Brazilian network research of the *Eucalyptus* Genome (Genolyptus) was established with the goal of integrating several academic and private institutions currently working with eucalyptus genomics in Brazil. This project generated around 200.000 ESTs from several tissues and conditions. Consequently, several individual projects have been implemented generating other transcriptome databases, in special, using RNA-Seq technology. In 2010, a draft genome (http://eucalyptusdb.bi.up.ac.za) of the specie *E. grandis* was produced by researches of the Joint Genome Institute (DOE-JGI) and the *Eucalyptus* Genome Network (EUCAGEN). The main goal of this work is to develop an *Eucalyptus*database (http://www.lge.ibi.unicamp.br/genolyptus) integrating public and private data in a friendly and secure web interface with bioinformatics tools that allowing the users perform complex searches.

## Results and discussion

First, the public and private ESTs (130,290 from Genolyptus and 36,981 from NCBI) were assembled producing 48,760 unigenes (17,795 contigs and 30,765 singlets). Basically, the bdtrimmer [1] and CAP3 [2] programs were used to perform sequence trimming (exclude vector, ribosomal, low quality and too short reads) and sequence assembly, respectively.

The autofact pipeline [3] was used to perform an automatic annotation of the assembled unigenes based on BLAST [4] searches, e-value cutoff of 1e-5, against some protein databases, including: non-redundant (NR) database of NCBI, uniref90 and uniref100 – databases containing only curated proteins [5], pfam – database of proteins families [6], kegg – database of metabolic pathways [7] and Gene Ontology (GO) – database of functional annotation [8].

The Genomic and Expression Laboratory at State University of Campinas (http://www.lge.ibi.unicamp.br) sequenced ten RNA-Seq libraries from four species (*E. Urograndis*, *E. globulus*, *E. grandis* and *E. urophylla*) using the Illumina/Solexa technology. Additionally, three RNA-seq libraries [9] were downloaded from NCBI (SRA – sequence read archive). All RNA-seq reads were aligned against the assembled unigenes and genome assembly using the SOAP2 [10] and TopHat [11] aligners, configured to allow up two mismatches, discard sequences with “N”s and return all optimal alignments.

In order to perform a differential expression analysis between ESTs or RNA-seq libraries some normalization pipelines and statistical tests have been implemented.From ESTs, the differentially expressed genes between libraries were performed applying AC test [12] in assembled unigenes. The results are available to the users by a web interface (called Electronic Northern) that allows searches by gene or library name. Furthermore, it is possible to compare the gene expression between two or more libraries. From the RNA-seq libraries, the DEG-seq software [13] was used to perform normalization and statistical analysis considering 99% of confidence rate (cut-off of 0.01).

**Figure 1:**
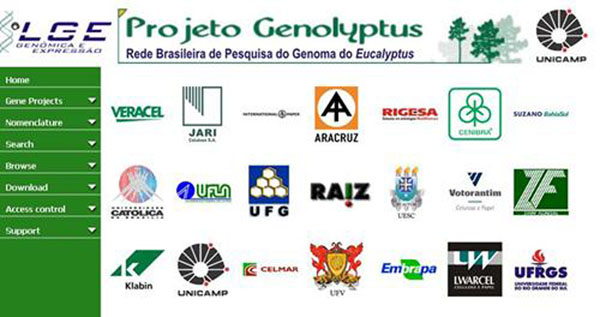
Home-page of the *Eucalyptus* database, hosted at http://www.lge.ibi.unicamp.br/genolyptus.

**Figure 2:**
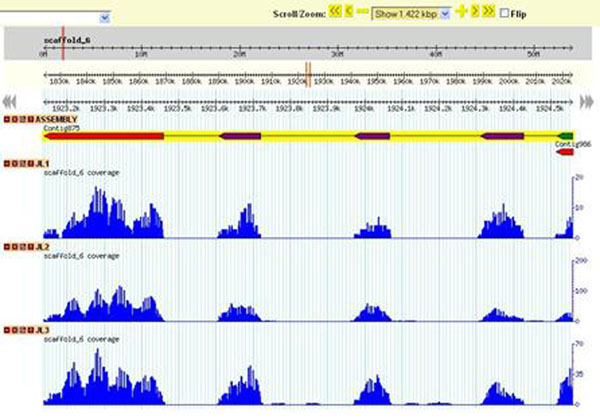
Gbrowse interface of the *Eucalyptus* database. Using Gbrowse is possible to compare gene expression between the RNA-Seq libraries.

To integrate all data described above, we developed a web site (Fig. 1) hosted in a Fedora Linux machine with MySQL database server. The web interface is based on a combination of CGI scripts using PERL language (including BioPerl module) and the Apache Web Server. The site contains many bioinformatics tools allowing the user perform keyword or local BLAST search in assembled unigenes. Also it is possible to connect these results with gene expression analysis. Moreover, the Gbrowse software (Generic Genome Browser) (Fig. 2) was used to visualization the data in a genomic context, integrating the different information by clickable tracks. The top track is the reference genome assembly and the other tracks correspond to assembled unigenes and RNA-seq data mapped into reference.
